# The control and importance of hyaluronan synthase expression in palatogenesis

**DOI:** 10.3389/fphys.2013.00010

**Published:** 2013-02-04

**Authors:** Jennifer L. Galloway, Sarah J. Jones, Peter A. Mossey, Ian R. Ellis

**Affiliations:** Unit of Cell and Molecular Biology, Dundee Dental School, University of DundeeDundee, UK

**Keywords:** HA, Has, palatogenesis, TGFβ, cleft palate

## Abstract

Development of the lip and palate involves a complex series of events that requires the close co-ordination of cell migration, growth, differentiation, and apoptosis. Palatal shelf elevation is considered to be driven by regional accumulation and hydration of glycosoaminoglycans, principally hyaluronan (HA), which provides an intrinsic shelf force, directed by components of the extracellular matrix (ECM). During embryogenesis, the extracellular and pericellular matrix surrounding migrating and proliferating cells is rich in HA. This would suggest that HA may be important in both shelf growth and fusion. TGFβ3 plays an important role in palatogenesis and the corresponding homozygous null (TGFβ3^−/−^) mouse, exhibits a defect in the fusion of the palatal shelves resulting in clefting of the secondary palate. TGFβ3 is expressed at the future medial edge epithelium (MEE) and at the actual edge epithelium during E14.5, suggesting a role for TGFβ3 in fusion. This is substantiated by experiments showing that addition of exogenous TGFβ3 can “rescue” the cleft palate phenotype in the null mouse. In addition, TGFβ1 and TGFβ2 can rescue the null mouse palate (*in vitro*) to near normal fusion. *In vivo* a TGFβ1 knock-in mouse, where the coding region of the TGFβ3 gene was replaced with the full-length TGFβ1 cDNA, displayed complete fusion at the mid portion of the secondary palate, whereas the anterior and posterior regions failed to fuse appropriately. We present experimental data indicating that the three HA synthase (Has) enzymes are differentially expressed during palatogenesis. Using immunohistochemistry (IHC) and embryo sections from the TGFβ3 null mouse at days E13.5 and E14.5, it was established that there was a decrease in expression of Has2 in the mesenchyme and an increase in expression of Has3 in comparison to the wild-type mouse. *In vitro* data indicate that HA synthesis is affected by addition of exogenous TGFβ3. Preliminary data suggests that this increase in HA synthesis, in response to TGFβ3, is under the control of the PI3kinase/Akt pathway.

## Introduction

Palatogenesis is a developmental event which starts at week 4 in human embryonic development and is not completed until around week 12 (Mossey et al., 2009); and is comprised of two main events i.e. formation of the primary and the secondary palate. The focus of this study will be the events implicated in formation of the secondary palate. The palatal shelves grow in a vertical orientation following the influx of mesenchymal cells at the maxillary process. There are two particularly important events in secondary palatogenesis: palatal shelf elevation and palatal shelf fusion (Mossey et al., 2009). Historically, it was believed that during palatal shelf elevation in mice, the epithelial area at the tip of the vertical palatal shelf would rotate above the tongue to become the medial edge epithelium (MEE) (Gritli-Linde, 2007). However, alternative methods have also been muted such as, a “flowing” movement (Greene and Kochhar, 1973; Diewert and Tait, 1979) and the production of an outgrowth from the side of the palatal shelf so that the MEE is composed of epithelial cells from the edge of the palatal shelves nearest the tongue (Jin et al., 2010). It has also been suggested that the most anterior and posterior region of the palate have a different method of changing palatal shelf orientation than the mid-palate. It is reported that the mid-palate elevates by rotation and remodeling the medial edge of the shelf, in accordance with the thoughts of Jin et al. (2010), while the front and back portions of the palate develop new tissue by remodeling. Once the shelves have elevated, they grow toward each other, the shelves touch and form a mid-line epithelial seam which then disappears to form the completely fused secondary palate (Mossey et al., 2009).

Cleft palates are a common developmental abnormality with around 1–2 per 1000 births affected (Christensen, 2002). Unfortunately, the molecular events involved in palatogenesis are currently poorly understood, thus there are no clear ways of preventing the development of oral clefts. It is therefore essential that research into the mechanisms involved in abnormal palatogenesis is carried out, as a cleft palate can have a marked affect a patient's quality of life (Marcusson et al., 2001).

The TGFβ family consists of more than 30 proteins (Nawshad et al., 2005). These proteins are involved in numerous biological processes including, proliferation, differentiation, and apoptosis, as well as epithelial-mesenchymal transition (EMT), extracellular matrix (ECM) synthesis and deposition (Martinez-Alvarez et al., 2000). There are three human isoforms; TGFβ1, TGFβ2, and TGFβ3, which are all expressed in the epithelium of the palatal shelves at different stages of palatogenesis (Meng et al., 2009). Although the isoforms are known to share approximately 71–76% sequence homology, their roles in embryogenesis (and after) vary significantly (Rotzer et al., 2001). Their specific function is highly dependent upon which member of the TGFβ family is activated. For example, for EMT, TGFβ1 activation occurs in cancer, while activation of TGFβ3 occurs during development (Yu et al., 2009).

Hyaluronan (HA) is a high molecular mass polysaccharide consisting of D-glucuronic acid (1-β3) N-acetyl D-glucosamine (1-β4) disaccharide repeats (Ellis et al., 1997). Unlike other glycosaminoglycans (GAGs), HA is not synthesized within the Golgi apparatus, but at the plasma membrane by the addition of UDP-Glucuronic acid and UDP-N-acetyl glucosamine on membrane bound enzymes known as HA synthases (Has) (Moffatt et al., 2011). These enzymes synthesize HA and secrete it directly through the plasma membrane into the extracellular environment (Moffatt et al., 2011). The Has family is comprised of three members; Has1, Has2, and Has3, which are all composed of a transmembrane domain and a large cytosolic catalytic domain (Laurent and Fraser, 1992; Weigel et al., 1997; Moffatt et al., 2011). However, they differ in their stability, in the time they are expressed during development (Tien and Spicer, 2005), their level of expression in different cell types, with growing cells expressing higher levels than those that are not (Jacobson et al., 2000) and the way they are affected by external stimuli (Ellis et al., 2007). Each of the Has produces HA with the same structure, but with various molecular weights, thus allowing cells to utilize HA differently (Itano et al., 1999) (Table 1). As the Has enzymes are important in many processes during and following development, they must be strictly regulated. This may occur post-transcriptionally by inhibition of translation by naturally occuring anti-sense Has2 (Nishida et al., 2005; Michael et al., 2011), dimersation of the Has enzymes, modification of the enzymes through monoubiquitination, the ABC transport system which moves HA into the ECM and the effect of certain lipids making up the plasma membrane (Karousou et al., 2010). Furthermore, regulation may occur through increased or decreased levels of the sugar substrates needed for HA production (Ventura et al., 2006). The rate at which the enzymes are translated may also be important (Recklies et al., 2001).

**Table 1:** **Differences between the Has enzymes**.

	**Has1**	**Has2**	**Has3**
Gene Location in Humans (Spicer et al., 1997)	Boundary of 19q13.3 and 19q13.4	8q24.12	16q22.1
Gene Location in Mice (Spicer et al., 1997)	Chromosome 17	Chromosome 15	Chromosome 8
Expression-mRNA (Tien and Spicer, 2005)	During gastrulation. Stopped by E8.5 in mice	At all times during development. The only Has expressed in the elevating palatal shelves	After E10.5 in mice. The only Has present in the dental epithelial placode
Stability (Tien and Spicer, 2005)	Least stable	Intermediate stability	Most stable
Molecular Weight of HA Produced (Itano et al., 1999)	Between 2 × 10^5^ and 2 × 10^6^ Da	Between 2 × 10^5^ and 2 × 10^6^ Da	Between 1 × 10^5^ and 1 × 10^6^ Da

Previous studies have reported a link between HA and TGFβ3 in wound healing. The roles of HA and TGFβ3 have also been linked to scarless wound healing. Longaker et al. (1991), suggest that HA may be vital in providing foetal cells with an environment for scarless wound healing, as it is present for much longer in foetal cells compared to the short initial presence in adult cells. Furthermore, HA may be able to prevent or reduce adult scar formation through exogenous application (Hellstrom and Laurent, 1987; Shah et al., 1995) speeding up the process of wound healing (Laurent and Fraser, 1992). It could therefore be possible that a common pathway involving TGFβ3 and HA is involved in scarless wound healing which is a throwback to the early link in developmental processes such as secondary palate formation.

Our working hypothesis is that HA has a role in normal palatogenesis both in terms of hydration, enabling shelf elevation and as a matrix for cell migration and proliferation. Perturbation of growth factors such as TGFβ3 and EGF lead to reduced synthesis of HA (or changes in the size of HA produced) and can lead to the formation of a cleft palate.

## Materials and methods

### Immunohistochemistry (IHC)

Coronal tissue sections of wild-type and TGFβ3 null mouse embryo crania (C57 strain), fixed in 4% (v/v) paraformaldehyde, were a gift from Professor M. Dixon (Manchester Dental School). After fixing and pre-treatment with citrate buffer (pH6) at 95°C, the tissue sections were stained using a method based on that described in Jones et al. (2007). Normal swine serum (20% v/v in PBS, Vector) was used to block non-specific staining in sections to be stained for Has1 and Has2 and normal goat serum (20% v/v in PBS, Vector) was used to block sections to be stained for Has3. Following optimization, the Has1, Has2, and Has3 primary antibodies (sc23145, sc34068, sc66917, Santa Cruz Biotechnology) were used at a concentration of 2 μg/ml. Biotinylated swine anti-goat (DakoCytomation, Glostrup, Denmark) and biotinylated goat anti-rabbit (Vector, Burlingame, USA) secondary antibodies were used at concentrations of 1:150 and 1:166 respectively. Primary antibody only, secondary antibody only and blocking peptides for sc34068 and sc66916 (Santa Cruz Biotechnology) were used as negative controls. Staining was visualized using an avidin-biotinylated enzyme complex (Vectastain ABC kit, Standard Elite, Vector Labs.) and incubation with DAB (3′,3′-diaminobenzidine), a substrate for the enzyme. Sections were counter-stained with haematoxylin.

### 3D collagen gel migration assay

Type I collagen from rat tail tendons was used to make 2 ml collagen gels in 35 mm plastic tissue culture dishes as previously described (Ellis et al., 2010). Collagen gels were overlaid with 1 ml of either serum-free MEM (SF-MEM) or SF-MEM containing 4 × the final concentration of the test compounds to be examined. Confluent stock cultures of fibroblasts were then harvested, resuspended in growth medium containing 4% (v/v) donor calf serum at 2 × 10^5^ cells/ml and 1 ml aliquots were added to the overlaid gels. Considering the 2 ml volume of gel, 1 ml medium overlay and 1 ml cell inoculums, this procedure gives a final concentration of 1% (v/v) serum in both control and test cultures. Fibroblasts attach to the surface of the gel within 1 h and start migrating into the underlying 3D gel within 24 h. Four days after plating, the number of cells that remain on the surface or migrate into the gel was determined by microscopic observation of 10 randomly selected fields in each of duplicate cultures (Ellis et al., 2010). Cell migration was then expressed by the number of cells that migrated into the 3D gel, as a percentage of the total number of cells present (mean ± SEM).

### Metabolic labeling and determination of total labeled HA

GAGs synthesized by adult and foetal fibroblasts were metabolically labeled by incubating cultures with 2.5 μCi ml^−1^ of [^3^H]-glucosamine for 4 days. Metabolically labeled cell cultures were then separated into medium and pericellular fractions (Ellis et al., 1992). For cells plated onto collagen gels, the medium and gel were transferred to a centrifuge tube and spun at 3000 rpm for 20 min at 4°C. The medium was removed and the compacted gel resuspended in 2 ml of phosphate-buffered-saline (PBS). Centrifugation was repeated and the supernatant was then combined with the previous one, to form the medium fraction. The pericellular fraction was obtained by extracting the cell layer with 2 ml 4M-guanidinium chloride, 50 mM-Tris, pH 7.4, for 24 h at 4°C. The insoluble material was removed by centrifugation at 12,000 g for 2 min in a microcentrifuge and the supernatant was collected as the pericellular fraction. The medium and pericellular fractions were dialyzed against 0.1 M sodium acetate, 5 mM EDTA, pH 5.5 for 48 h (with two changes per day). For cells plated on plastic tissue culture dishes, the medium was transferred to a centrifuge tube. The dish was then washed with 2 × 1 ml PBS which was added to the medium. The cells on the dish were extracted in the same manner as the cells on the collagen gel, as described above.

The dialysate volumes were measured and six 200 μl aliquots were taken of each sample. 20 μl of hyaluronate lyase (final concentration 0.5 U/ml in acetate buffer) was added to three of these, whilst 20 μl of acetate buffer was added to the three remaining controls. All samples were incubated for 17 h at 37°C, then boiled for 1 min to inactivate the enzyme and unlabeled carrier GAG added (30 μl 1% chondroitin sulphate/0.5% HA in PBS). The undigested GAGs were precipitated with 4 volumes of 1.3% potassium acetate in 100% ethanol at −20°C for 3 h (Underhill and Toole, 1979). The precipitate was sedimented by centrifugation at 12,000 g for 5 min, the supernatant was again removed and the pellet redissolved in 100 μl of 1 M sodium hydroxide and boiled for 3 min. The sodium hydroxide was neutralized with 100 μl of 1 M hydrochloric acid. The GAGs were transferred to scintillation vials had 1 ml of scintillant added and the radioactivity determined using a Packard scintillation counter. The disintegrations per minute (DPM) associated with HA was taken as the difference between the counts in the control and enzyme digested preparations and were controlled per 10^5^ cells.

A relatively insignificant proportion (<5%) of the total synthesized HA was found in the pericellular fraction under all experimental conditions examined; data are consequently only presented for HA present in the medium fraction.

### SDS PAGE

Cell lysates from treated fibroblasts were made with RIPA buffer using the method described in Klemke et al. (1994) and separated by 8% SDS PAGE, under reducing conditions (Laemmli, 1970). Samples were mixed with Laemmli loading buffer (Bio-Rad Laboratories Ltd., Hemel Hempstead, Hertfordshire UK) containing 5% (v/v) 2-mercaptoethanol and heated for 5 min at 95°C prior to loading onto the gel. Magic Markers (Invitrogen Ltd.) were also loaded.

### Western blotting

SDS PAGE gels were electroblotted onto nitrocellulose at 15 V for 42 min, using a Bio-Rad semi-dry blotting apparatus and the transfer buffer 48 mM Tris, 39 mM glycine, 1.3 mM SDS, 20% v/v methanol (Towbin et al., 1979). Buffers used during development of the blots were based on TBS (20 mM Tris-HCl, 137 mM sodium chloride pH 7.6). Blots were blocked in blocking buffer (1% w/v low fat skimmed milk powder, 0.05% v/v Tween 20, in TBS) for 10–30 min at room temperature and then incubated overnight at room temperature in blocking buffer, containing anti-phospho-Akt (Ser473) antibody (Cell Signaling Technology). Blots were then washed (3 × 20 min) in TBST (TBS, 0.05% v/v Tween 20) and incubated in a 1:2000 dilution of goat anti-rabbit HRP (Cell Signaling). Blots were then washed (3 × 20) min in TBST and finally for 5 min in TBS. Bands were then detected by chemiluminescence using the substrate SuperSignal West Dura (Pierce, Thermo Fisher Scientific).

### Immunocytochemistry

Cell cultures were incubated overnight in serum-free MEM and on the following day placed in test conditions for 1 h prior to fixation. Medium was removed from the dishes and cells were rinsed with TBS. Cells were incubated in 4% v/v paraformaldehyade at 4°C for 20 min, then washed with TBS for 5 min, three times. Cells were then washed in 0.2% Triton for 5 min and TBS again for 5 min, three times. Cells were then left in 3% H_2_O_2_ at room temperature for 20 min then washed in TBS twice for 5 min. Areas of the dish were circled with an immunopen and 150 μl 5% v/v Normal Goat Serum was added to each ring for 30 min. The dishes were rinsed and washed twice with TBS for 5 min. Each ring was treated with 150 μl primary antibody [phospho Akt (Ser473) Cell Signaling Technology] or negative controls and incubated in a humidified chamber at 4°C overnight. On the following day dishes were equilibrated to room temperature for an hour before being rinsed in TBS and washed twice in TBST for 5 min. Cells were washed for 5 min in TBS before addition of the detecting system (Signal Boost from Cell Signaling), 1–3 drops per ring. After 30 min, dishes were rinsed with TBS, washed twice in TBST for 5 min, then TBS for 5 min. Cells were incubated in DAB solution for 10 min at room temperature. To stop the reaction, dishes were rinsed in tap water before counterstaining with Mayer's Haematoxylin for 30 s and Bluing Agent for 1 min. Coverslips were mounted using aqueous mount and plates viewed using a Motic BA400 microscope.

## Results

### Localization of Has1, Has2, and Has3 *ex vivo* studies of developing embryonic mouse palate

**Figure 1:**
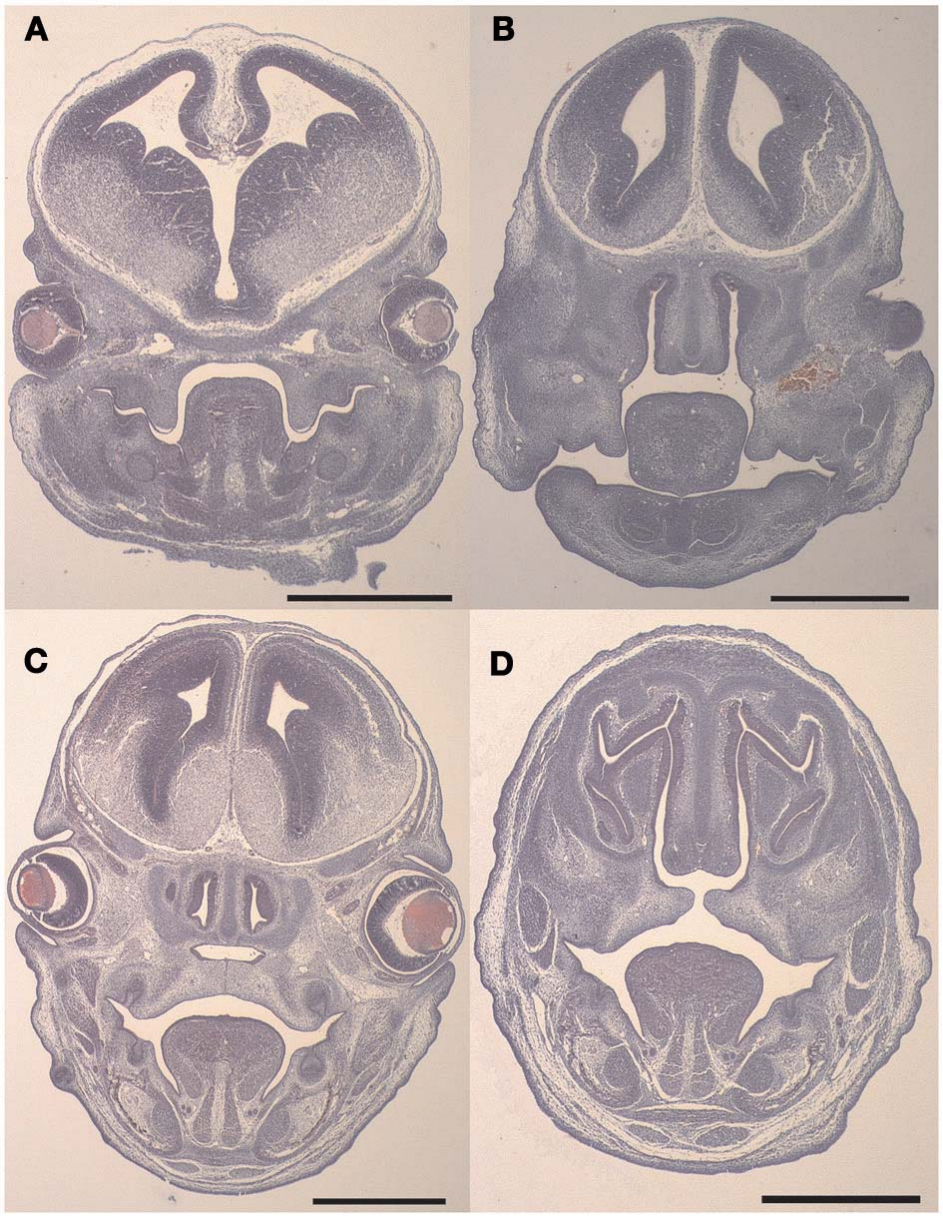
**Coronal sections of C57 Wild-type and TGFβ3 null mouse crania stained with H&E before and after palatal shelf elevation.** Representative coronal sections of wild-type and TGFβ3 null mouse crania at embryonic ages E13.5 and E14.5 stained with H&E are provided for orientation purposes **(A–D)**. The wild-type mouse sections are **(A)** (E13.5) and **(C)** (E14.5) and the TGFβ3 null mouse sections are **(B)** (E13.5) and **(D)** (E14.5).

**Figure 2:**
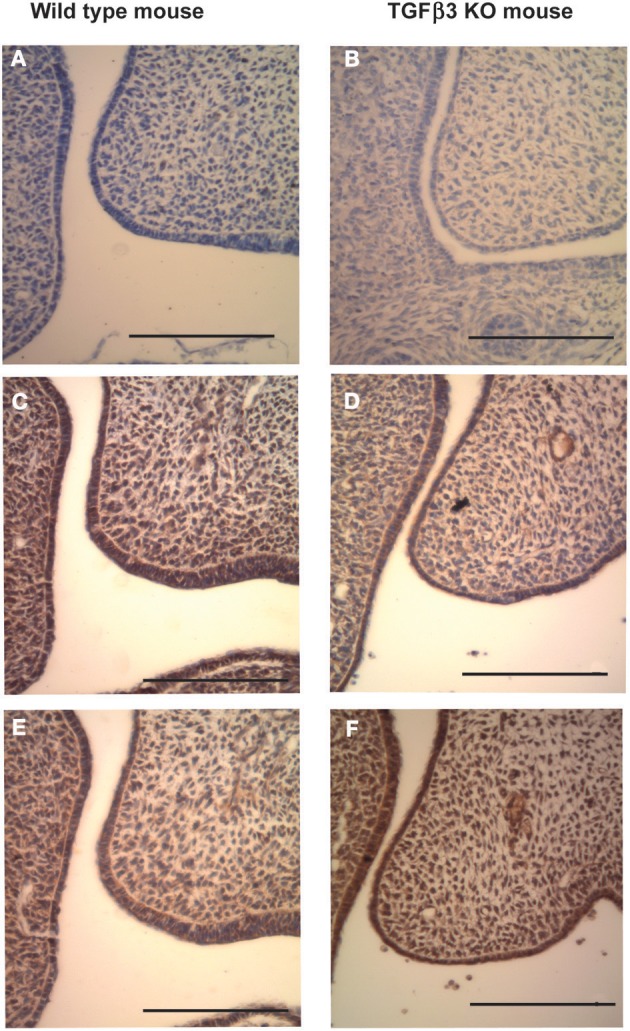
**Coronal sections of C57 Wild-type and TGFβ3 null mouse crania stained for Has expression before palatal shelf elevation (E13.5).** Wild-type (**A,C** and **E**) and TGFβ3 null (**B,D** and **F**) mouse palatal shelves at E13.5 were stained for Has1 using a polyclonal goat anti-Has1 antibody **(A)**, wild-type mouse and **(B)**, TGFβ3 null mouse, Has2 using a polyclonal goat anti-Has2 antibody **(C)**, wild-type mouse and **(D)**, TGFβ3 null mouse or Has3 using a polyclonal rabbit anti-Has3 antibody **(E)**, wild-type mouse and **(F)**, TGFβ3 null mouse. Positive staining appears brown whereas negative staining appears purple. The bars on the diagrams represent 200 μm. Both wild-type and TGFβ3 null mouse palatal shelves appeared to have low expression for Has1. Whereas there is a slight reduction in Has2 expression and a slight increase in Has3 expression between the wild type and the TGFβ3 homozygous null mouse.

The first process of secondary palate development is shelf elevation. In the wild-type or TGFβ3 null C57 strain of mouse this takes place around E13.5 *in utero*. We investigated the expression of the Has enzymes just prior to shelf elevation. Neo-natal mouse heads were collected from E13.5 embryos either having the wild-type or the TGFβ3 homozygous null phenotype. The H and E sections indicate that the shelves had not elevated in both the wild-type and knockout mouse heads (Figures 1A and B). These were fixed, paraffin-embedded, sectioned and stained, according to the method describe above, with antibodies raised against Has1, Has2, and Has3 (Figures 2A–F). In the wild-type, there was very little or no staining for Has1 in the sections at E13.5. Staining for Has2 was intense around the epithelium and there was some staining in the mesenchyme. Has3 was intensely stained around the epithelium with strong staining in the mesenchyme. In the TGFβ3 null mouse, staining for Has1 was negative. Staining for Has2 indicated that there was some present in the epithelium and mesenchyme but was reduced in amount. Staining for Has3 was increased in both the epithelium and the mesenchyme compared to the wild-type.

**Figure 3:**
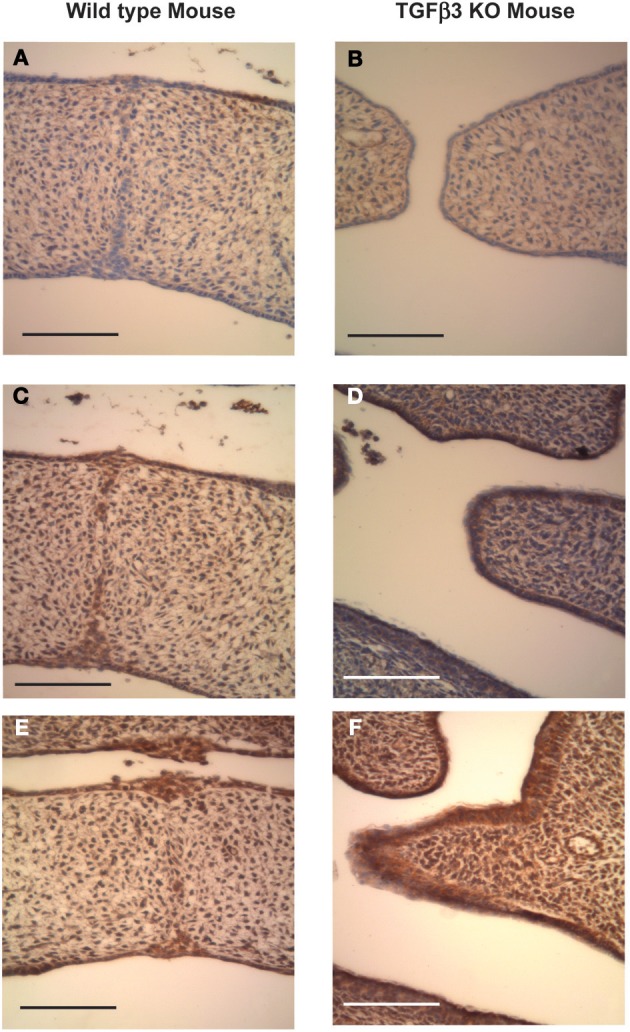
**Coronal sections of C57 Wild-type and TGFβ3 null mouse crania stained for Has expression after palatal shelf elevation (E14.5).** Wild-type (**A,C** and **E**) and TGFβ3 null (**B,D** and **F**) mouse palatal shelves at E14.5 were stained for Has1 using a polyclonal goat anti-Has1 antibody **(A)**, wild-type mouse and **(B)**, TGFβ3 null mouse, Has2 using a polyclonal goat anti-Has2 antibody **(C)**, wild-type mouse and **(D),** TGFβ3 null mouse or Has3 using a polyclonal rabbit anti-Has3 antibody **(E)**, wild-type mouse and **(F)**, TGFβ3 null mouse. Positive staining appears brown whereas negative staining appears purple. The bars on the diagrams represent 200 μm. The expression of Has1 and 2 appears reduced in the absence of TGFβ3, with the reduction in Has2 being the greatest. Has3 expression may be slightly increased in the absence of TGFβ3.

The second major event of secondary palatogenesis is fusion of palatal shelves (Figures 1C and D). This usually takes place in the developing mouse around day 14.5 *in utero*. We have therefore investigated the expression of the Has enzymes as the wild-type palates fuse. It should be noted that in the TGFβ3 null C57 strain of mouse embryos, the shelves did not meet (Figures 3A–F). Neo-natal mouse heads from both phenotypes were collected and were sectioned and stained with antibodies raised against Has1, Has2, and Has3. In the wild-type mouse, there was some staining of Has1, especially in the mesenchyme and some in the epithelium although it was absent in the epithelial triangles. Has2 expression appears similar to that at E13.5 appearing in both the mesenchyme and the epithelium. It appears quite intense around the disappearing midline epithelium. The expression of Has3 is very strong both in the epithelium and the mesenchyme and more intense than Has2 staining in the epithelium. In the TGFβ3 null mouse, the palatal shelves do not meet, Has1 staining is very low and staining for Has2 was reduced in both the epithelium and the mesenchyme. Has3 appears to be expressed quite strongly especially when compared with Has1 and Has2.

### *In vitro* studies of foetal and adult fibroblasts: response to TGFβ3

**Figure 4:**
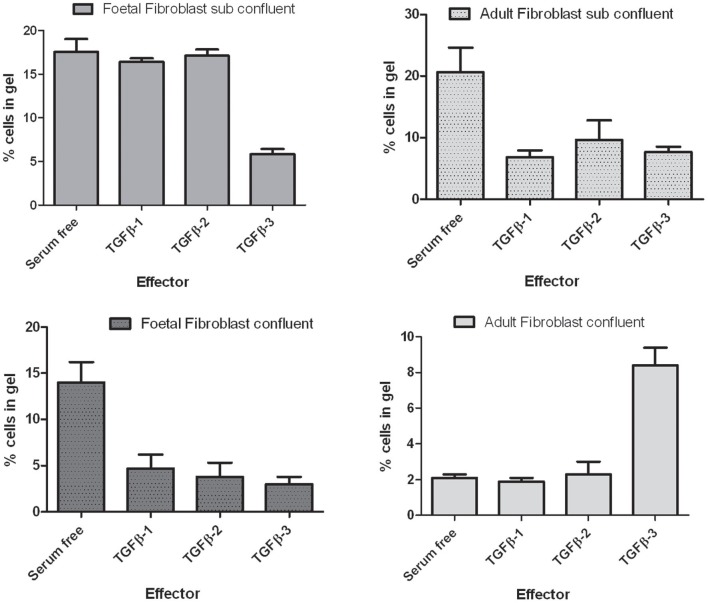
**The effects of TGFβ isoforms on the migration of foetal and adult skin fibroblasts.** Summary of the data obtained with three lines of both foetal and adult skin fibroblasts. Cells were plated onto collagen gels at subconfluent and confluent cell densities. Cells were incubated with concentrations of the TGFβ isoforms which elicited then maximal response (0.1 ng/ml TGFβ3 on confluent adult fibroblasts and 10 ng/ml TGFβ2, and 3 under all other conditions). The number of cells that had migrated into the collagen matrix after 4 days of incubation was determined microscopically. Data are expressed as the percentage of cells within the gel matrix.

**Figure 5:**
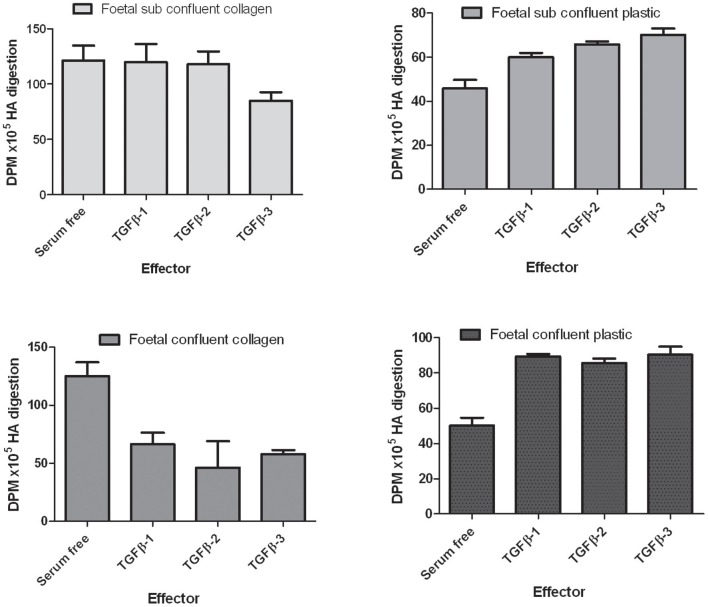
**The effects of TGFβ isoforms on HA synthesis by foetal fibroblasts.** Summary of the data obtained with three lines of foetal fibroblasts. Cells were plated onto either collagen gels or plastic tissue culture dishes at subconfluent and confluent cell densities. Cells were incubated with 10 ng/ml of each of the TGFβ isoforms for 4 days. Data are expressed as [^3^H]-disintigrations per minute × 10^5^ /10^5^ cells incorporated into HA, determined as described in Ellis and Schor (1998).

The reduction of Has protein expression in the mouse palate in the TGFβ3 null mouse lead to the development of our hypothesis that HA is important in palatogenesis. Previous studies using the mouse C57 strain suggests that loss of functional TGFβ3 protein always produces a cleft palate (Proetzel et al., 1995). The interaction between TGFβ3, cell migration and HA synthesis has been reported in the literature with respect to adult and foetal fibroblasts (Ellis and Schor, 1998) but their possible interaction has not been investigated in the context of palatogenesis. The exact pathway involving TGFβ3 (and other TGFβ family members) in palatogenesis is currently unknown, but previous *in vitro* studies have found that TGFβ3 affects the production of HA in human fibroblasts (Ellis and Schor, 1998). During palatal fusion a number of mechanisms have been reported to be important in the disappearance of the mid-line including EMT, apoptosis and migration. However, which one of these mechanisms is most important is open to debate. Our investigations have led us to study the effect of growth factors on cell motility. Cell migration is also affected by the addition of different TGFβ proteins to fibroblasts. Fibroblasts from both foetal and adult origin were isolated in the laboratory and were characterized by their ability to respond to various members of the TGFβ family. The addition of different TGFβ3 isoforms to fibroblasts affects cell motility depending upon the confluence of the cells and their origin (Figure 4). TGFβ3 inhibits the migration of both adult and foetal fibroblasts plated onto the surface of 3D collagen gels at sub-confluent cell densities. However, when plated at confluent cell density, foetal cells are inhibited and adult cells stimulated to migrate into the collagen gel. Foetal fibroblasts produce different amounts of HA *in vitro* (Figure 5) and the addition of TGFβ isoforms to foetal skin fibroblasts on collagen gels has been shown to inhibit HA synthesis but stimulate HA synthesis when plated on normal tissue culture dishes (Figure 5).

**Figure 6:**
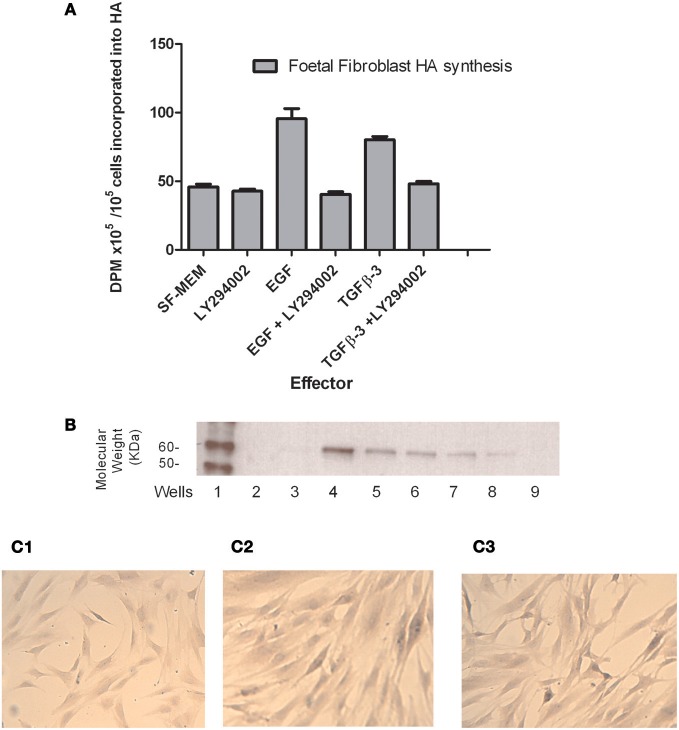
**The effects of inhibitor LY294002 on foetal fibroblasts in response to EGF and TGFβ3. (A)** HA synthesis: foetal fibroblasts were plated on plastic tissue culture dishes at confluent cell density in the presence of either 10 ng/ml EGF or 0.1 ng/ml TGFβ3 and/or 1 μM LY294002. Data are expressed as [^3^H]-glucosamine incorporated into HA as determined by digestion with Hyaluronan lyase. **(B)** SDS-PAGE and Western blotting of TGFβ3 activation of phospho- Akt (Ser 473) phosphorylation in foetal fibroblasts. Foetal fibroblasts were plated onto plastic tissue culture dishes. When confluent they were serum starved and on the following day placed into test medium. After 15 min the cells were lysed and run on an 8% SDS-PAGE. Proteins were blotted and probed with antibody against phospho- Akt (Ser 473). Lane 1 markers, Lane 2 Blank, Lane 3 SF-MEM, Lane 4 10%FCS, Lane 5 10 pg/ml TGFβ3, Lane 6 100 pg/ml TGFβ3, Lane 7 1 ng/ml TGFβ3, Lane 8 10 ng/ml TGFβ3, Lane 9 100 ng/ml TGFβ3. **(C)** Foetal fibroblasts plated onto 60 mm tissue culture dish. After 24 h the cells were placed in serum free overnight. The following day they were placed in test conditions for 1 h and fixed and stained for p473 Akt. **(C-1)** serum free control; **(C-2)** 0.1 ng/ml TGFβ3 **(C-3)** 0.1 ng/ml TGFβ3 and 1 μM LY294002.

The pathways by which cells respond to growth factors were investigated *in vitro*. Using a foetal fibroblast line the effect of growth factors on HA synthesis was investigated. The amount of HA synthesized in response to both EGF and TGFβ3 was increased. Experiments employing a number of signal transduction pathway inhibitors found that the PI-3 kinase pathway was important for HA synthesis (data not shown). Addition to the assay of the inhibitor LY294002 with EGF and TGFβ3 inhibited HA synthesis to background levels (Figure 6A). The PI-3 kinase pathway signals via the protein Akt, which is important in cell migration, EMT and apoptosis. The addition, to the cells, of increasing amounts of TGFβ3 increased Akt phosphorylation at Serine 473 which fully activates Akt (Figure 6B). Immunocytochemistry of fixed cells indicated that the phosphorylated protein appears to translocate to the nucleus when compared with control cells (Figures 6C–1 and C–2). Treatment of the cells with the inhibitor LY294002 reduced the amount of pAkt staining and also reduced the amount translocated to the nucleus (Figure 6C–3).

## Discussion

The data presented indicates: (1) that the palatal shelves of the TGFβ3 homozygous null strain of mice elevated as normal. (2) The palatal shelves of the TGFβ3 homozygous null mouse did not meet and therefore could not fuse. (3) Has1 staining appeared to be present only during the fusion process of the wild-type mouse and was absent in the TGFβ3 homozygous null mouse. (4) Has2 staining was present during both the elevation and fusion processes of the wild-type mouse but reduced in the TGFβ3 null mouse. (5) Has3 staining was present during both phases of palatal development in the wild-type mouse but only slightly increased in the TGFβ3 null mouse. (6) The TGFβ family of proteins affects cell migration in a cell density and cell origin dependent manner. (7) The TGFβ family of proteins affects HA synthesis in a matrix dependent manner. (8) TGFβ3 uses the PI3kinase/akt pathway to affect HA synthesis.

### Hyaluronan and its role in palatogenesis

The study of Has expression in the normal palate suggests that Has2 and 3 are expressed in the shelves prior to elevation and that HA is synthesized by both the epithelium and mesenchyme. In normal palate development it has been proposed that the main intrinsic force for elevation is provided by HA (Ferguson, 1988), which mechanically traps large quantities of water in its large branched network structure (Scott et al., 1991), thus causing expansion of the palatal shelves and aiding elevation. Our data confirm that in normal palate development, HA can be synthesized in the palatal shelves and does not have to be transported from other areas. It should be noted that the shelves also elevate in the TGFβ3 homozygous null mouse. Our data suggests that there is a reduction in the amount of Has2 and Has3 present in these shelves. It may be that even a small amount of these enzymes can produce enough HA for the shelves to elevate. However, the reduced expression of these enzymes at this time point may effect development at a later stage, for example when the palatal shelves are growing toward one another before fusing. Has2 synthesizes high molecular weight HA (Itano et al., 1999) which has different properties to the lower molecular weight HA synthesized by Has3. Growth factors have been reported to increase the amount of high molecular weight HA synthesized by mesenchymal cells from both foetal and adult origins (Ellis et al., 1997, 1999). The ECM that cells are grown on can also affect response to growth factors and this could explain the fact that TGFβ3 increases HA synthesis of foetal fibroblasts plated on plastic tissue culture dishes but decreases it when the cells are plated on collagen gels. This increase in high molecular weight HA synthesis can be accompanied by an increase in cell migration. Addition of high molecular weight HA increases the migration of adult fibroblasts into 3D collagen gels suggesting that the high molecular weight HA has roles to play in tissue development other than binding of large amounts of water. High molecular weight HA when added to fibroblasts plated onto the surface of 3D collagen gel causes an increase of migration into the gel (unpublished). HA can also influence cell behavior and development through interactions with other molecules, including the HA cell surface receptor Cluster of Differentiation 44 (CD44) (Aruffo et al., 1990) and as part of a pericellular matrix structure (Laurent and Fraser, 1992). Finally, the molecular weight and concentration of previously synthesized HA located in the ECM may influence further HA production (Smith and Ghosh, 1987). At a transcriptional level, regulation of Has activity may occur at regions upstream of each of the Has genes through stimulation by cytokines and other factors (Monslow et al., 2003), such as, epidermal growth factor (EGF) (Yamada et al., 2004), transforming growth factor α (TGFα) (Bachem et al., 1989) and members of the transforming growth factor β (TGFβ) family (Sugiyama et al., 1998).

### TGFβ3 and its role in palatogenesis

TGFβ3 has been found to have a vital role in palatogenesis as TGFβ3 homozygous null mice develop a cleft palate due to a failure in the fusion process (Proetzel et al., 1995), while the exogenous addition of TGFβ3 can reverse cleft palate development in mice (Shah et al., 1995). In palatogenesis, TGFβ3 is the first member of the TGFβ family to be expressed and it exhibits spatial expression within the palatal shelves, which changes as development progresses (Fitzpatrick et al., 1990). The absence of TGFβ3 does not affect the development of any other part of the craniofacial region in mice, which could suggest that the effect of TGFβ3 on palatogenesis involves a primary, intrinsic mechanism (Proetzel et al., 1995).

The exact role of TGFβ3 in palatogenesis remains unclear with conflicting literature published on its role in adhesion of the opposing shelves. It may also have an important role in allowing the cell movement requisite for fusion (Proetzel et al., 1995; Kaartinen et al., 1997; Martinez-Alvarez et al., 2000). TGFβ3 homozygous null mouse models have been very important in developing an understanding of the processes involved in palatogenesis. In general development, TGFβ3 is involved in EMT as well as regulating a large number of cell processes including, cell proliferation, cell differentiation and the production of ECM (Miettinen et al., 1994). However, it is highly plausible that there are complex interactions between many growth factors, which may explain why different strains of TGFβ3 homozygous null mice develop different phenotypes of cleft palate (Proetzel et al., 1995). Many interactions have been suggested in the literature, for example, TGFβ3 may regulate palatal shelf adhesion through chondroitin sulphate proteoglycans, which appear on the apical surface of MEE cells just before the shelves meet (Gato et al., 2002). It has also been suggested that TGFβ3 upregulates lymphoid-enhancing factor 1 (LEF1) using Smad2,4/LEF1, which in turn allows EMT to occur (Nawshad and Hay, 2003). Furthermore, Xu et al. (2006), suggest that the transcription factor Irf6 is controlled by TGFβ and that it could have an important role in the regulation of both human and mouse MEE cells during fusion. TGFβ family members are able to affect cell processes by utilizing type I, type II threonine kinase receptors and Smad proteins to affect DNA transcription (Massague, 1998). The signals produced by these receptors must be strictly regulated (Gritli-Linde, 2007). The role of the TGFβ type II receptor in palatogenesis is emphasized by the development of a cleft palate in mouse embryos lacking the TGFβ type II receptor gene (Xu et al., 2006). In addition to the SMAD pathway, TGFβ3 has been reported to activate the PI3kinase/akt pathway (Nawshad and Hay, 2003). Preliminary *in vitro* data described here suggests that this may be linked to HA synthesis. Previous work has reported that the phosphorylation of Akt is important for cell motility (Ellis et al., 2010). The data reported here indicate that this pathway is up-regulated in response to mesenchymal cells to TGFβ3 and that blocking of this pathway also affects HA synthesis. It remains to be determined if this response is a direct or an in direct response of this pathway.

Many studies have focused on a number of human populations to establish whether the role of TGFβ3 in cleft palate development can be applicable to humans. These studies report conflicting results, with both positive relationships (Maestri et al., 1997; Romitti et al., 1999; Mitchell et al., 2001; Sato et al., 2001; Beaty et al., 2002; Jugessur et al., 2003; Kim et al., 2003; Vieira et al., 2003; Ichikawa et al., 2006; Suazo et al., 2010) and no relationship reported (Lidral et al., 1997; Tanabe et al., 2000; Beaty et al., 2001; Suzuki et al., 2004). Thus, it could be that TGFβ3 is of importance in cleft palate development but only in certain populations.

## Conclusion and future work

**Figure 7:**
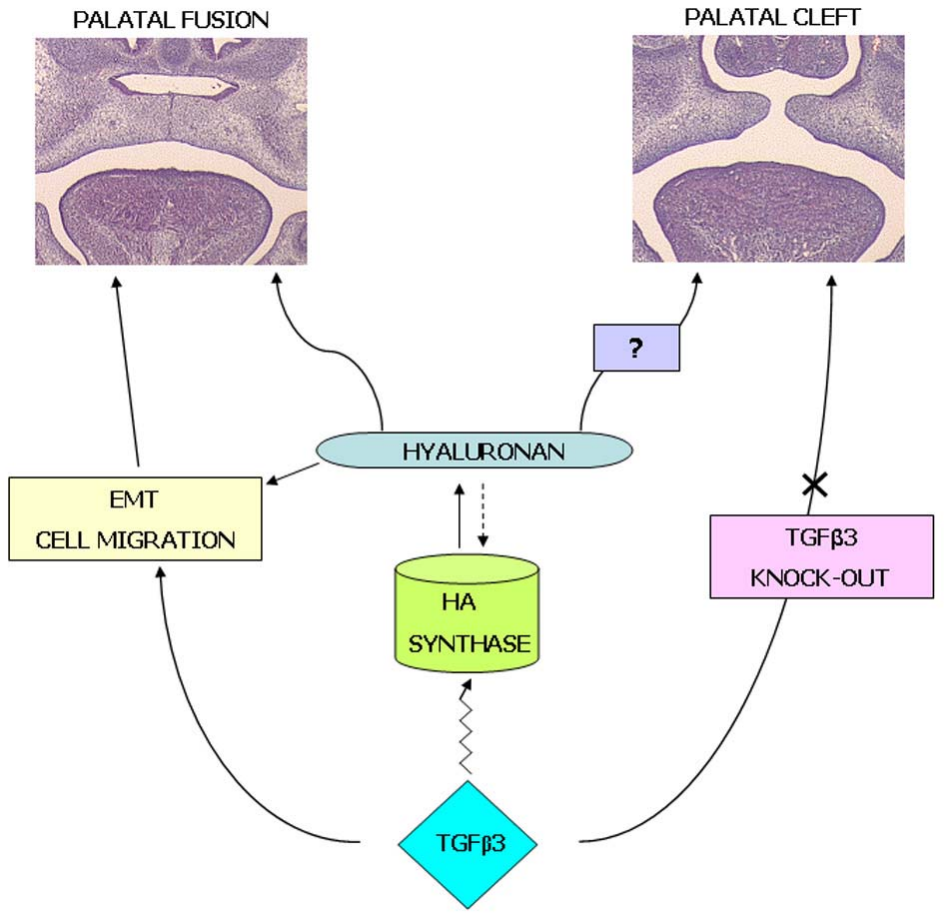
**Hypothesis: the interaction between TGFβ3 and HA is interlinked in palatogenesis.** Our working hypothesis is that HA has a role in normal palatogenesis both in terms of hydration enabling shelf elevation and as a matrix for cell migration and proliferation. Perturbation of growth factors such as TGFβ3 and EGF lead to reduced synthesis of HA (or changes in the size of HA produced) and can lead to the formation of a cleft palate.

The mechanisms that regulate shelf elevation are very important as once the palatal shelves have changed orientation, they must meet in the midline of the embryo in preparation for fusion. It is likely that HA is involved in the increase in length of the palatal shelves required for them to meet, and thus it could follow that molecules involved in fusion have a role in regulating HA synthesis. Many molecular factors have been suggested as having a role in fusion, especially those with adhesion qualities, with a review by Gritli-Linde (2007), listing over 70 genetic mutations that have been shown to produce cleft palates. The interaction between HA and TGFβ3 is summarized in our hypothesis (Figure 7), where HA has a multi-functional role as a substrate that can bind water, growth factors/proteins, cells and allows for normal development to take place. However, changes in HA expression or its role can lead to the development of cleft palate.

Future experiments will be to investigate Has expression in the same strain of TGFβ3 homozygous null mouse at a later point in development to assess whether the palatal shelves do eventually meet in the midline. The link between Has expression and TGFβ3 in the MF1 strain mouse, where the palatal shelves meet in the midline but do not fuse, will also be studied. This will establish whether there is a difference in Has expression in different phenotypes of cleft palate, which have developed due to absence of TGFβ3.

The expression of Has2 can be modulated by exogenous factors. We are in the process of creating a series of cell lines where the expression of TGFβ3 has been silenced by shRNA. This will enable examination, *in vitro*, of the factors and mechanisms controlling Has2 synthesis and also environmental factors such as alcohol and nicotine. This *in vitro* model is currently being employed to study the signaling pathways important in TGFβ3 activation of the TGFβ receptors.

### Conflict of interest statement

The authors declare that the research was conducted in the absence of any commercial or financial relationships that could be construed as a potential conflict of interest.
